# The iceLogo web server and SOAP service for determining protein consensus sequences

**DOI:** 10.1093/nar/gkv385

**Published:** 2015-04-20

**Authors:** Davy Maddelein, Niklaas Colaert, Iain Buchanan, Niels Hulstaert, Kris Gevaert, Lennart Martens

**Affiliations:** 1 Department of Medical Protein Research, VIB, A. Baertsoenkaai 3, B-9000 Ghent, Belgium; 2 Department of Biochemistry, Faculty of Medicine and Health Sciences, Ghent University, A. Baertsoenkaai 3, B-9000 Ghent, Belgium

## Abstract

The iceLogo web server and SOAP service implement the previously published iceLogo algorithm. iceLogo builds on probability theory to visualize protein consensus sequences in a format resembling sequence logos. Peptide sequences are compared against a reference sequence set that can be tailored to the studied system and the used protocol. As such, not only over- but also underrepresented residues can be visualized in a statistically sound manner, which further allows the user to easily analyse and interpret conserved sequence patterns in proteins. The web application and SOAP service can be found free and open to all users without the need for a login on http://iomics.ugent.be/icelogoserver/main.html.

## INTRODUCTION

The development of high throughput methods for analysing oligonucleotides and proteins led to the discovery of large amounts of sequence-based information. These data can contain conserved sequence patterns which may explain specificities of the studied processes. In 1990 Schneider and Stephens described a method to visualize and analyse such conserved patterns (1). These so-called sequence logos are histogram-like presentations where every bar is a stack of letters (being amino acids or nucleotides) and are created with a group of sequences of the same length as the input. The height of a stack is calculated by Shannon's information theory. This takes into account the maximum number of possible different residues (4 different nucleotides or 20 different amino acids) and the observed frequencies of these residues on that position in the experimental multiple sequence alignment. The size of one residue in such a stack thus reflects the frequency of this residue at a given position. A web-based application, WebLogo, implements this sequence logo algorithm (2).

Despite its overall usefulness and wide adoption by the scientific community, this method has two major shortcomings. First, the experimental set is not compared with a reference set. This means that the reference is implicitly assumed to be a fixed and equal contribution (25% for a nucleic acid and 5% for an amino acid) for every residue, and this clearly does not reflect reality. Slogos (oligonucleotides) and Plogos (proteins) attempt to address this issue by providing the user with the ability to set a fixed frequency for every residue, and so create a corrected sequence logo still relying on Shannon's information theory (3). Second, while over-represented residues in a consensus sequence are clearly visible in a sequence logo, the equally important underrepresented residues in a consensus sequence are not at all visualized and are therefore readily overlooked. If two experimental sets are available, the differences in both over- and under-represented residues can however be visualized by the TwoSampleLogo web application (4). Several other methods also adapt sequence logos to better visualize specific aspects of oligonucleotide or protein sequence patterns (5–10). The iceLogo algorithm however, not only resolves these problems but also creates additional, complementary visualizations that ease the analysis of protein consensus sequences (11).

## IceLogoServer

Here we present the implementation of the iceLogo algorithm in a web server and a SOAP service. The web application is designed to make the creation of rich and precise iceLogo visualizations very easy for users, while the SOAP service is aimed at developers who want to transparently implement the iceLogo algorithm in their own software.

## ALGORITHM

Below we describe the functionality of the iceLogo server, but a full description of the iceLogo algorithm can be found in reference (11) and in the online manual.

Two ways of creating a reference set are implemented: a static and a dynamic reference set. While the static method is available in both the web application and the SOAP service, the more complete but more complex dynamic method can only be used via the SOAP service. The static method takes either a list of reference sequences as input, thus providing different residue frequencies for different positions or can be selected from pre-calculated, species-specific proteome frequencies, yielding identical residue frequencies for each position in the alignment. The dynamic method on the other hand performs a Monte Carlo sampling strategy to create a reference set on-the-fly from a species-specific FASTA protein database. The amino acids or peptides can be randomly extracted from the FASTA database, or can be derived following more complex methods, including sampling peptides at a specific position. This for instance allows sampling from only terminal peptides (i.e. peptides within a certain distance from the amino or carboxy terminus of the protein) that are proven to have a different composition than internal peptides (12). The algorithm will then calculate significances (*Z*-scores) for the amino acids in the experimental set using the frequencies of the amino acids in the reference set and the sample size obtained. The results can then be visualized in different ways, as detailed below.

### VISUALIZATIONS

**Figure 1.**
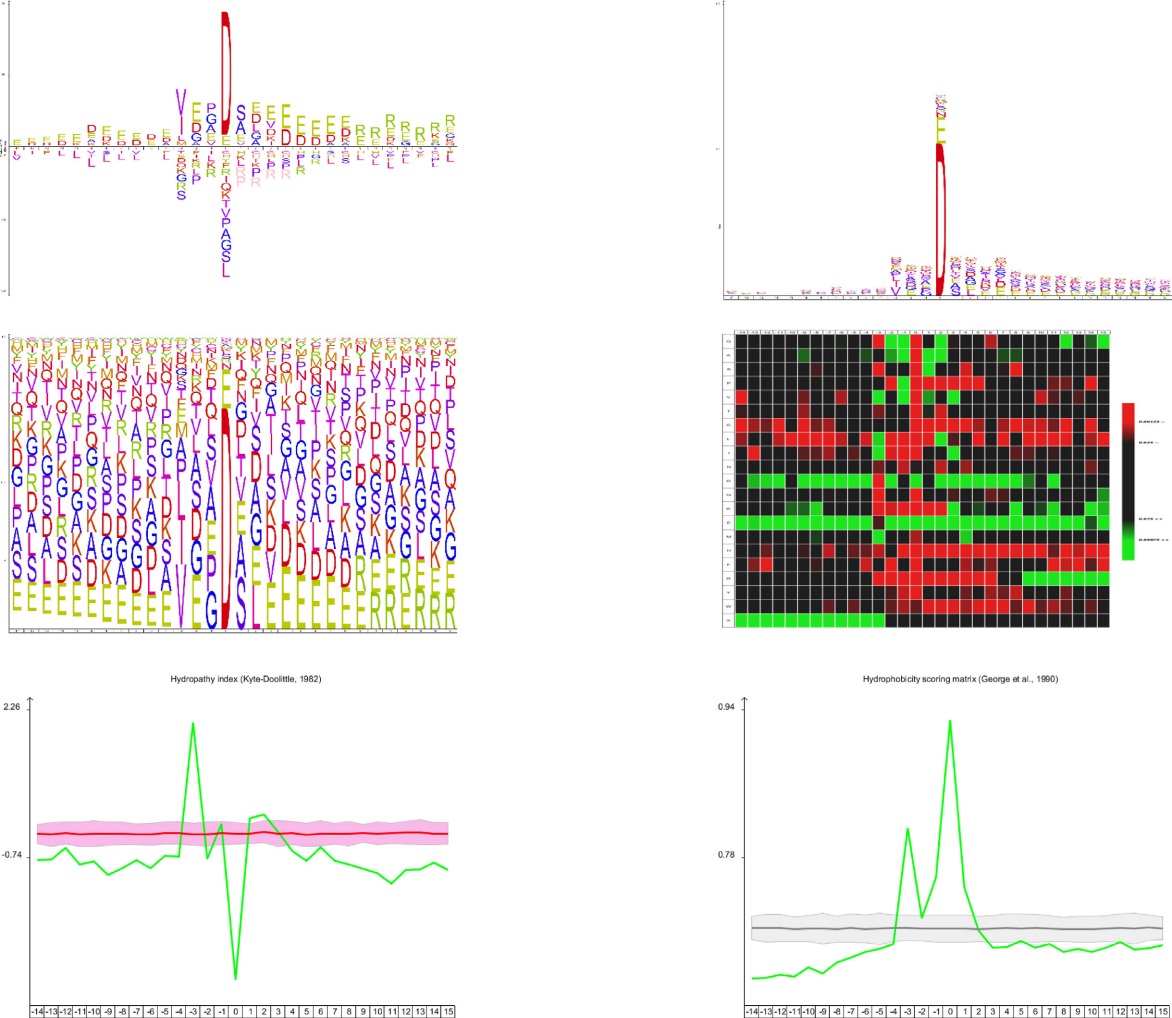
123 substrates of the mouse granzyme B protease (that cleaves at the carboxyl-terminus of an aspartate) are used the display the versatility of visualization methods supported by the web server and the SOAP service. The processing site is shown as an iceLogo (upper left corner), as a corrected sequence logo (upper left corner), as a filled sequence logo (middle left), as a heat map (middle right), as an amino acid parameter graph displaying the hydropathy of the residues (lower left corner) and a correlation graph showing the consensus hydrophobicity index. The human subset of the UniProtKB/Swiss-Prot database was used to calculate amino acid frequencies for the reference set. These different visualization methods clearly provide more detailed information concerning the processing site than a sequence logo alone.

Six different visualizations exist that provide comprehensive and complementary views on the available information. The iceLogo plot attempts to visualize a consensus sequence in a rich and precise manner similar to sequence logos, but with two changes: the use of a reference set allows iceLogos to rely on probability to find and visualize only significantly different residue frequencies in the experimental set and iceLogo also provides the visualization of significantly underrepresented residues, indicating non or less tolerated residues in the consensus sequence. These latter residues are plotted below the abscissa in the iceLogo. The second visualization is a corrected sequence logo, similar to the output of Plogos (3). Indeed, since iceLogo can extract position-specific frequencies from the reference set, the sequence logo height at every position can be corrected with the actual sequence bias at that location. The third visualization is a variant of the normal sequence logo. The entire graph space is used to represent the amino acids at each position. The amino acids themselves are represented as their percentual abundance on each given location. This reduces the impact of a heavily up or downregulated amino acid on a certain position in favour of the relative impact of each amino acid on each location. The fourth visualization provided by the tool is a heat map view that shows all amino acid occurrences and significances for all positions in a single image. The heat map is drawn as a two-dimensional matrix in which every row represents a residue and every column a position. Every cell in this matrix is coloured according to the representation of the residue at that position: a cell is black if it is not significantly represented, or a shade of green or red for significantly up or down-represented residues, respectively. The fifth visualization displays specific amino acid factors like charge and hydrophobicity (or any other physicochemical or biochemical parameter of the 544 possibilities from the AAIndex 1 database (13)) in an amino acid parameter graph. This graph thus visualizes a common parameter in the context of the reference set. Finally, the sixth visualization is parallel with the previous and visualizes the correlation between a substitution matrix and the positional amino acids. An example of each of the six different visualizations is given in Figure 1, where 123 mouse granzyme C cleavage sites are compared with the mouse proteome as a static reference set (14).

## AVAILABILITY

The iceLogo web application can be found at http://iomics.ugent.be/icelogoserver/main.html. The intuitive design of the web page should enable users to quickly become acquainted with its interface. The only obligatory input is a list of sequences that are expected to share residue-related features. The reference set can be created by specifying a list of reference sequences or via the selection of a species-specific proteome constructed from the UniProtKB/Swiss-Prot protein database (15). Other parameters concerning the visualization type, colour of the residues, *P*-value etc. can be set before generating the visualization. The online manual provides various samples covering the different visualization methods and adjustable parameters that can serve as a guide to users. The created visualizations can be viewed and downloaded in various image file formats (JPEG, TIFF, PNG, PDF and SVG).

The SOAP service can be programmatically accessed via the SOAP protocol on http://iomics.ugent.be/icelogoserver/services/icelogo. A WS-I complaint document/literal-wrapped WSDL file describing the various methods of the SOAP service can be found on http://iomics.ugent.be/icelogoserver/IceLogo.wsdl. Additionally, the available methods and their parameters are also explained on the iceLogo website. The SOAP service generates the iceLogo results as lightweight, xml based SVG images. Both the iceLogo algorithm and the IceLogoServer are published under the permissive Apache 2 open source licence (http://www.apache.org/licenses/LICENSE-2.0.html) and the source is available via git from https://github.com/compomics/icelogo and https://github.com/compomics/icelogoserver, respectively. A preassembled web archive (WAR) file can also be found on the latter website, making it very easy to create a local, customized iceLogo web application or SOAP service if desired. For most users however, we recommend using the well-maintained and fully tested web server described here. A sample Java SOAP client is briefly described on the website and more examples (including an implementation of a client that converts SVG to JPEG, TIFF, PNG or PDF images) can be found in the iceLogoServer code.

## USAGE STATISTICS

The iceLogo web server has been available online continuously since 2010. The recent usage statistics provided in Table 1 highlight the popularity of the web service. The JPEG export format is clearly the most popular, with PNG and PDF taking up the second and third place. TIFF and SVG are less popular, despite the usefulness of these formats for inclusion in publications.

**Table 1.** iceLogo website usage statistics in average number of iceLogos created per month, split by generated image type

	JPEG	PNG	SVG	TIFF	PDF	Total
Average number of generated iceLogos per month	558	72	23	40	52	746

Statistics are calculated over the 15-month period from October 2013 up to and including December January 2014.

## CONCLUSION

The web application and SOAP service presented here implement a stable and popular online version of the iceLogo algorithm. The web application allows the user to create comprehensive protein consensus sequence visualizations easily in an intuitive web environment, thus bypassing the hassle of downloading and installing a local program. Due to the platform and language independency of the SOAP architecture, bioinformaticians can use the iceLogo visualization in their own software without the need to use the java library containing the iceLogo algorithm.
